# Relationship between Body Roundness Index and cognitive impairment in middle-aged and older adults: a population-based cross-sectional study

**DOI:** 10.3389/fnagi.2025.1522989

**Published:** 2025-02-19

**Authors:** Dandan Guo, Tao Li, Qingchao Yang, Chenlu Yang, Yang Yang, Fuchen Liu, Jun Ma, Jun Tu, Xianjia Ning, Jinghua Wang, Chengyuan Song, Yiming Liu

**Affiliations:** ^1^Department of Neurology, Qilu Hospital of Shandong University, Jinan, Shandong, China; ^2^School of Bioengineering, Shandong Polytechnic, Jinan, Shandong, China; ^3^Department of Geriatrics, Qilu Hospital of Shandong University, Jinan, Shandong, China; ^4^Department of Neurology, Tianjin Medical University General Hospital, Tianjin, China; ^5^Laboratory of Epidemiology, Tianjin Neurological Institute, Tianjin, China; ^6^Key Laboratory of Post-Neuroinjury Neuro-Repair and Regeneration in Central Nervous System, Ministry of Education and Tianjin City, Tianjin Neurological Institute, Tianjin, China

**Keywords:** Body Roundness Index, cognitive impairment, obesity, rural population, cross-sectional study

## Abstract

**Background:**

Cognitive impairment is a growing public health concern, particularly in aging populations. Obesity, as measured by various indices, has been linked to cognitive decline, but the relationship between Body Roundness Index (BRI) and cognitive impairment remains unclear. This study aims to evaluate the association between BRI and cognitive impairment in a rural, low-income, low-education population in China and to determine if BRI can be used as an independent predictor of cognitive decline.

**Methods:**

This cross-sectional study included the participants aged 35–95 years from rural Tianjin, China. The mean age of the study population was 64.35 ± 7.58 years. Data were collected through face-to-face interviews, physical examinations, and laboratory tests. Cognitive function was assessed using the Mini-Mental State Examination (MMSE), and BRI was calculated and grouped into quartiles. Univariate and multivariate logistic regression analyses were performed to examine the relationship between BRI and cognitive impairment. Subgroup analyses were conducted to explore interactions between BRI, age, gender, and hypertension. The dose–response relationship was analyzed using restricted cubic spline models.

**Results:**

Of the participants, 36.5% had cognitive impairment. Multivariate analysis showed that women, individuals aged 65 and over, and those with hypertension had a higher risk of cognitive impairment. Participants in the second quartile of BRI had a 31% lower risk of cognitive impairment compared to the first quartile (OR: 0.69, 95% CI: 0.51–0.94, *p* = 0.017). Subgroup analysis revealed that BRI was significantly associated with cognitive impairment in individuals under 65, but not in older participants. The dose–response relationship between BRI and MMSE score showed an inverted U-shaped curve, with the weakest association observed around a BRI of 4.49.

**Conclusion:**

Body Roundness Index, in conjunction with age, gender, and hypertension, can serve as a useful predictor of cognitive impairment, particularly in younger populations. Early identification of individuals at risk through BRI may facilitate timely interventions, reducing the burden of cognitive decline on patients and healthcare systems.

## 1 Introduction

Cognitive impairment, including dementia, presents a significant global health challenge, with Alzheimer’s disease (AD) being the most common cause of dementia in individuals aged 65 and older. By 2024, approximately 6.9 million Americans aged 65 and above will have Alzheimer’s disease, a figure projected to rise to 13.8 million by 2060 (Tahami Monfared et al., 2022; Alzheimer’s Association, 2024). In China, the incidence of AD among the elderly reached 600.49 per 100,000 in 2019, with an annual growth rate of 1.12% (Li et al., 2024). Neurological disabilities such as AD and other dementias ranked among the top 10 contributors to disability-adjusted life years (DALYs) in 2021(GBD 2021 Nervous System Disorders Collaborators, 2024). The Global Burden of Disease study reported that in 2021, AD and dementia accounted for 1.3% of all health-related losses, resulting in 11.6 million years lost to disability (YLDs), a 45.7% increase over a decade (GBD 2021 Diseases and Injuries Collaborators, 2024). The economic impact is also staggering; it is estimated that the cost of long-term care for dementia patients aged 65 and over will reach $360 billion by 2024 (Alzheimer’s Association, 2024). Given these alarming trends, identifying and mitigating risk factors for cognitive impairment at an early stage is crucial.

Body Roundness Index is a novel anthropometric measurement that better reflects body fat percentage and visceral adipose tissue (Thomas et al., 2013). It also serves as an alternative marker for insulin resistance (IR) (Feng et al., 2019). Obesity, a global epidemic, is projected to affect nearly half of the world’s adult population by 2030 (Neto et al., 2023). China, in particular, faces the highest prevalence of overweight and obese individuals worldwide (Wang et al., 2021). Research has demonstrated that obesity and its related metabolic disturbances—such as oxidative stress, inflammation, and IR—contribute to neuronal damage, brain atrophy, and subsequent cognitive decline (Neto et al., 2023; Dye et al., 2017; Li et al., 2023). For instance, visceral fat area (VFA) has been negatively associated with delayed memory, language scores, and MMSE scores (Moh et al., 2020; Ozato et al., 2021). Additionally, VFA is linked to cognitive decline in men, while subcutaneous fat area (SFA) correlates with cognitive decline in women (Uchida et al., 2024). Intriguingly, greater fat mass and lean body mass are associated with a reduced risk of cognitive impairment in older women (Noh et al., 2017).

Despite these findings, studies specifically investigating the relationship between BRI and cognitive impairment remain scarce. A cross-sectional study in Taiwan suggested a significant inverse relationship between BRI and MMSE scores in individuals over 60 years (Huang et al., 2022).Similarly, a US population study reported a significant negative correlation between BRI levels and cognitive performance in individuals over 65 years of age (Zhang et al., 2024). However, other studies, including those conducted in rural China and Iran, found no significant association between BRI and dementia or cognitive performance (Wang et al., 2023; Ramezani Kashal et al., 2024). These conflicting results, mostly focused on individuals over 60, highlight the need for further investigation. In this context, our study aims to fill this research gap by exploring the relationship between BRI and cognitive function in a younger, low-education population aged 35–95 in rural China.

The primary objective of this study is to assess the association between BRI and cognitive impairment in a socioeconomically disadvantaged, low-education population in rural China, using a population-based cross-sectional design.

## 2 Methods

### 2.1 Study population

The cross-sectional study analyzed data from 2,577 individuals collected from 18 rural areas in Tianjin, China, between 2012 and 2020, all of whom had low income and low educational attainment. The data were obtained through the Tianjin Brain Study, an ongoing, prospective, community-based cohort study. Participants were recruited from the local community, and data collection involved face-to-face interviews and physical examinations conducted by trained researchers at local community healthcare centers.

Participants with a history of cerebrovascular diseases, including cerebral infarction, transient ischemic attack, cerebral hemorrhage, and subarachnoid hemorrhage, were excluded to investigate the relationship between BRI and non-vascular cognitive impairment, minimizing confounding effects from vascular cognitive impairment. Additionally, those lacked height and waist circumference data were excluded.

**Figure 1:**
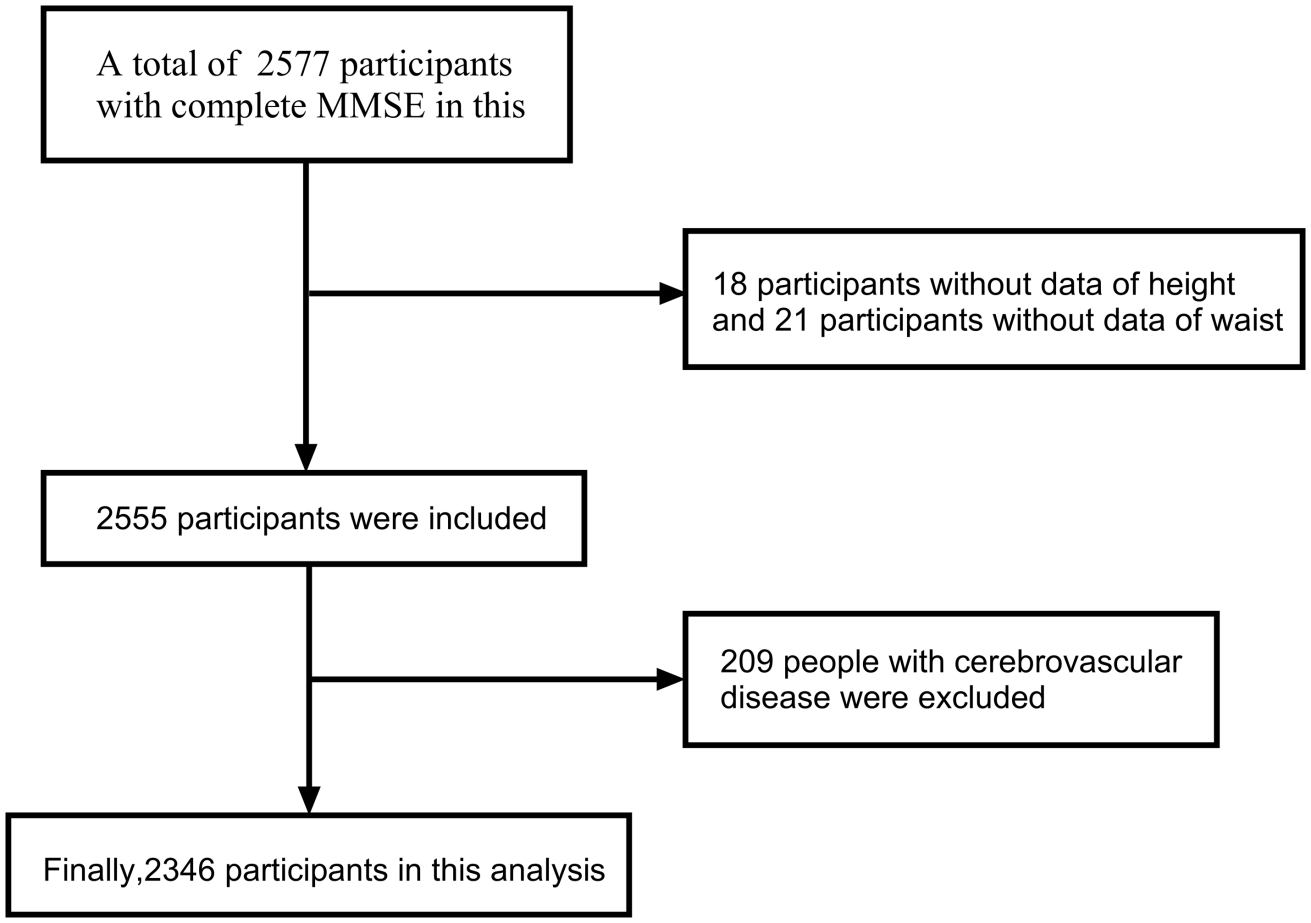
The flow chart of participants selection.

A total of 2,346 participants were included in the final analysis. The detailed selection process is illustrated in the flow chart (Figure 1). The study adhered to the principles of the Declaration of Helsinki and received approval from the Ethics Committee of Tianjin Medical University General Hospital. All participants provided written informed consent before participating in the study.

### 2.2 Data collection

Demographic and clinical data were gathered through face-to-face interviews conducted by trained researchers. The information collected included participants’ name, gender, age, years of education, and medical histories such as diabetes, hypertension, cerebrovascular disease, and myocardial infarction. Laboratory data, including triglyceride (TG), total cholesterol (TC), fasting blood glucose (FBG), and creatinine levels, were also obtained. Physical measurements were conducted using standardized procedures. Weight was measured with a standard scale, height was recorded with the participant standing upright, and waist circumference (WC) was measured using a soft tape at the midpoint between the iliac crest and the lowest rib. To minimize measurement errors, all measurements were performed by the same researcher.

### 2.3 Definitions and grouping

Hypertension was defined as systolic blood pressure (SBP) ≥140 mmHg and/or diastolic blood pressure (DBP) ≥90 mmHg, or a self-reported history of hypertension and current use of antihypertensive medication (Mancia et al., 2023). Diabetes was defined according to established criteria: hemoglobin A1c (HbA1c) ≥6.5%, fasting plasma glucose (FPG) ≥126 mg/dL (7.0 mmol/L), or a 2-h plasma glucose level ≥ 200 mg/dL (11.1 mmol/L) during an oral glucose tolerance test (OGTT), or a self-reported history of diabetes or use of hypoglycemic medication (American Diabetes Association Professional Practice Committee, 2024). Body mass index (BMI) was calculated as weight (kg) divided by height (m) squared, and participants were categorized as underweight (<18.5 kg/m^2^), normal weight (18.5 to <24 kg/m^2^), overweight (24 to <28 kg/m^2^), or obese (≥28 kg/m^2^) (Chen et al., 2023). BRI was calculated using the formula BRI = 364.2–365.5 × √(1 – [WC (cm)/2π]^2^/[0.5 × height (cm)]^2^) (Thomas et al., 2013), and participants were classified into quartiles based on BRI values (Q1, Q2, Q3, and Q4).

### 2.4 Cognitive function assessment

Cognitive function was evaluated using the MMSE, a widely applied tool for assessing cognitive decline (Chun et al., 2021). The MMSE assesses orientation, memory, attention and calculation, recall, and language ability, with scores ranging from 0 to 30. A lower score indicates worse cognitive function. A cohort study conducted in China has shown that MMSE scores are influenced by age, gender, and educational background. The cutoff points for cognitive impairment were set at ≤17 for illiterate participants, ≤20 for participants with 6 or fewer years of education, and ≤24 for participants with more than 6 years of education (Li et al., 2016).

### 2.5 Statistical analysis

Continuous variables were expressed as means with standard deviations (SD) or as medians with interquartile ranges (IQR) and were compared using Student’s *t*-test or the Mann–Whitney *U*-test, depending on the distribution of the data. Categorical variables were presented as frequencies and percentages and were compared using the chi-square test. Multivariate logistic regression analysis was employed to evaluate the association between BRI and other factors related to cognitive impairment. Participants with missing values for any variables included in the multivariate logistic regression analysis were excluded to maintain the validity and reliability of the results. To avoid collinearity, BRI and BRI quartile were included in separate multivariate logistic regression models. *β* values and their corresponding 95% confidence intervals (CI) were reported for all variables in the multivariate analysis.

Subgroup analyses were performed to further explore these relationships in different populations, based on variables identified in univariate analysis. In the subgroup analysis, multivariate logistic regression models were adjusted for key confounding factors, including age, gender, BMI, hypertension, and other relevant covariates such as diabetes and smoking history. The analysis aimed to explore the association between BRI and cognitive impairment within specific subgroups, controlling for these factors to assess their potential influence. The association between variables and cognitive impairment was expressed as adjusted odds ratios (OR) with 95% confidence intervals (CI). Restricted cubic spline (RCS) curves were used to investigate the dose–response relationship between BRI and cognitive impairment, with the number of spline nodes determined by the Bayesian information criterion (BIC). In the RCS plot, the bold curve represents the estimated regression coefficient, while the shaded area indicates the 95% CI. Statistical significance was defined as *p* < 0.05. All analyses were performed using SPSS version 27.0.1, and the flow chart and forest plots were created with GraphPad Prism version 10.2.3. R software (v.4.2) was used to generate the RCS curves.

## 3 Results

### 3.1 Demographic characteristics

Table 1 includes the total study population (*n* = 2,346), including participants with missing educational data. Characteristics are presented by gender for a comprehensive overview. A total of 2,346 participants were included in this study, comprising 1,021 males (43.5%) and 1,325 females (56.5%). The mean age of participants was 64.35 ± 7.58 years, with 78% of the population being between 60 and 79 years old. The average values for height, weight, waist circumference, BMI, BRI, and MMSE scores were 160.63 ± 8.01 cm, 65.08 ± 11.40 kg, 88.92 ± 10.18 cm, 25.14 ± 3.63 kg/m^2^, 4.50 ± 1.32, and 23 (IQR 7), respectively (Table 1).

**Table 1:** Demographic characteristics.

Characteristics	Man	Woman	Total
Total, *n* (%)	1,021 (43.5)	1,325 (56.5)	2,346 (100.0)
Age, years old	64.97 (7.53)	63.86 (7.58)	64.35 (7.58)
Age group, *n* (%)			
<65 years old	522 (51.1)	766 (57.8)	1,288 (54.9)
≥65 years old	499 (48.9)	559 (42.2)	1,058 (45.1)
Years of education, years^*^	6.13 (2.94)	3.65 (3.54)	4.72 (3.52)
Education Group, *n* (%)^*^			
Illiterate	60 (6.0)	456 (34.9)	516 (22.4)
Primary school	529 (53.3)	561 (42.9)	1,090 (47.4)
Junior school	330 (33.2)	231 (17.7)	561 (24.4)
High school and above	74 (7.5)	59 (4.5)	133 (5.8)
Height, cm	166.87 (6.14)	155.83 (5.61)	160.63 (8.01)
Weight, kg	69.06 (11.33)	62.02 (10.48)	65.08 (11.40)
Waist circumference, cm	89.21 (10.24)	88.69 (10.13)	88.92 (10.18)
BMI, kg/m^2^	24.72 (3.47)	25.46 (3.72)	25.14 (3.63)
BMI grouping, *n* (%)			
Normal or underweight	447 (43.8)	480 (36.2)	927 (39.5)
Overweight	392 (38.4)	545 (41.1)	937 (39.9)
Obesity	182 (17.8)	300 (22.6)	482 (20.5)
Smoking history, *n* (%)			
Never smoking	265 (26.0)	1,246 (94.0)	1,511 (64.4)
Current smoking	509 (49.9)	55 (4.2)	564 (24.0)
Ever smoking	247 (24.2)	24 (1.8)	271 (11.6)
Drinking history, *n* (%)			
Never drinking	372 (36.4)	1,279 (96.5)	1,651 (70.4)
Current drinking	535 (52.4)	30 (2.3)	565 (24.1)
Ever drinking	114 (11.2)	16 (1.2)	130 (5.5)
Hypertension, *n* (%)			
No	254 (24.9)	294 (22.2)	548 (23.4)
Yes	767 (75.1)	1,031 (77.8)	1798 (76.6)
Diabetes, *n* (%)			
No	874 (85.6)	1,052 (79.4)	1926 (82.1)
Yes	147 (14.4)	273 (20.6)	420 (17.9)
History of angina pectoris, *n* (%)			
No	996 (97.6)	1,299 (98.0)	2,295 (97.8)
Yes	25 (2.4)	26 (2.0)	51 (2.2)
History of myocardial infarction, *n* (%)			
No	1,018 (99.7)	1,321 (99.7)	2,339 (99.7)
Yes	3 (0.3)	4 (0.3)	7 (0.3)
Systolic blood pressure SBP, mmHg^*^	148.61 (22.77)	148.70 (23.36)	148.66 (23.10)
Diastolic pressure DBP, mmHg^*^	87.38 (11.59)	84.77 (11.94)	85.90 (11.86)
Fasting blood glucose FBG, mmol/L^*^	5.62 (1.53)	5.80 (1.73)	5.72 (1.65)
Total cholesterol TC, mmol/L^*^	4.44 (1.08)	4.79 (1.13)	4.64 (1.12)
Triglyceride TG, mmol/L^*^	1.58 (1.28)	1.77 (1.06)	1.69 (1.16)
Creatinine, μmol/L^*^	77.65 (27.74)	59.07 (11.80)	67.00 (22.19)
MMSE, M (IQR)	25 (6)	21 (8)	23 (7)
BRI	4.09 (1.16)	4.82 (1.34)	4.50 (1.32)
Quarter of BRI			
Q1	351 (34.4)	235 (17.7)	586 (25.0)
Q2	2,833 (27.7)	305 (23.0)	588 (25.1)
Q3	245 (24.0)	340 (25.7)	585 (24.9)
Q4	142 (13.9)	445 (33.6)	587 (25.0)

Table includes the total study population (*n* = 2,346), including participants with missing educational data. ^*^ indicates missing values, in which 46 cases are missing in education years and education groups, 14 cases are missing in FBG, 10 cases are missing in TG and 11 cases are missing in TC. There were 18 cases of SBP deletion, 19 cases of DBP deletion and 336 cases of creatinine deletion.

### 3.2 Associated factors of cognitive impairment in univariate analysis

After excluding 46 individuals due to incomplete education data, 2,300 participants were included in the analysis of cognitive impairment risk. Among them, 840 participants (36.5%) were found to have cognitive impairment. Univariate analysis revealed that several factors, including age, sex, height, weight, waist circumference, BMI, smoking history, drinking history, hypertension, SBP, FBG, creatinine, and BRI quartile, were significantly associated with cognitive impairment (*p* < 0.05) (Table 2).

**Table 2:** The associated factors of cognitive impairment in the univariate analysis.

Characteristics	No cognitive impairment	Have cognitive impairment	*t*/*χ*^2^	*P*
Total, *n* (%)	1,460 (63.5)	840 (36.5)	—	—
Gender, *n* (%)			44.924	< 0.001
Man	707 (71.2)	286 (28.8)		
Woman	753 (57.6)	554 (42.4)		
Age, years old	63.05 (6.93)	67.26 (7.22)	13.660	< 0.001
Age group, *n* (%)			81.990	< 0.001
<65 years old	897 (71.8)	352 (28.2)		
≥65 years old	563 (53.6)	488 (46.4)		
Height, cm	161.76 (7.91)	158.42 (7.69)	9.852	< 0.001
Weight, kg	66.31 (11.21)	62.59 (11.12)	7.667	< 0.001
Waist circumference, cm	89.57 (9.86)	87.62 (10.61)	4.363	< 0.001
BMI, kg/m^2^	25.26 (3.54)	24.86 (3.71)	2.543	0.011
BMI grouping, *n* (%)			6.490	0.039
Normal or underweight	550 (60.4)	360 (39.6)		
Overweight	597 (64.8)	324 (35.2)		
Obesity	313 (66.7)	156 (33.3)		
Smoking history, *n* (%)			40.996	< 0.001
Never smoking	863 (58.7)	608 (41.3)		
Current smoking	400 (71.4)	160 (28.6)		
Ever smoking	197 (73.2)	72 (26.8)		
Drinking history, *n* (%)			32.995	< 0.001
Never drinking	962 (59.7)	649 (40.3)		
Current drinking	408 (72.6)	154 (27.4)		
Ever drinking	90 (70.9)	37 (29.1)		
Hypertension, *n* (%)			12.792	< 0.001
No	362 (70.2)	154 (29.8)		
Yes	1,098 (61.5)	686 (38.5)		
Diabetes, *n* (%)			3.116	0.078
No	1,211 (64.3)	672 (35.7)		
Yes	249 (59.7)	168 (40.3)		
History of angina pectoris, *n* (%)			0.163	0.686
No	1,429 (63.5)	820 (36.5)		
Yes	31 (60.8)	20 (39.2)		
History of myocardial infarction, *n* (%)			0.119	0.730
No	1,456 (63.5)	837 (36.5)		
Yes	4 (57.1)	3 (42.9)		
Systolic blood pressure SBP, mmHg^*^	147.20 (22.70)	152.27 (23.36)	−5.085	< 0.001
Diastolic pressure DBP, mmHg^*^	86.13 (11.55)	85.81 (12.34)	0.631	0.528
Fasting blood glucose FBG, mmol/L^*^	5.66 (1.51)	5.84 (1.84)	−2.438	0.015
Total cholesterol TC, mmol/L^*^	4.63 (1.14)	4.65 (1.10)	−0.281	0.779
Triglyceride TG, mmol/L^*^	1.69 (1.19)	1.67 (1.12)	0.427	0.670
Creatinine, μmol/L^*^	68.04 (25.28)	64.78 (15.15)	3.071	0.002
BRI	4.50 (1.27)	4.50 (1.40)	−0.053	0.957
Quarter of BRI			14.761	0.002
Q1	336 (58.6)	237 (41.4)		
Q2	397 (68.7)	181 (31.3)		
Q3	374 (65.4)	198 (34.6)		
Q4	353 (61.2)	224 (38.8)		

^*^ indicates missing values, including FBG missing in 14 cases, TG missing in 10 cases and TC missing in 11 cases. There were 18 cases of SBP deletion, 19 cases of DBP deletion and 336 cases of creatinine deletion.

### 3.3 Association between BRI and cognitive impairment in multivariate analysis

Tables 3, 4 present the results of the multivariate logistic regression analysis. To address potential collinearity between BRI and BRI quartiles, two separate models were constructed. Model 1 (Table 3) included BRI as a continuous variable, while Model 2 (Table 4) used BRI quartiles. Both models were adjusted for age, gender, BMI, hypertension, and other relevant covariates. The *β* values, along with 95% confidence intervals (CIs), are reported for each variable.

**Table 3:** The association of BRI and cognitive impairment in the multivariate analysis.

Characteristics	Reference	*β*	OR (95%CI)	*P*
Gender	Man			
Woman		0.464	1.59 (1.12–2.25)	0.009
Age, years old	<65 years old			
≥65 years old		0.719	2.05 (1.69–2.50)	< 0.001
BMI grouping	Normal or underweight			
Overweight		−0.199	0.82 (0.63–1.06)	0.135
Obesity		−0.455	0.64 (0.43–0.94)	0.022
Hypertension	No			
Yes		0.368	1.45 (1.13–1.85)	0.004
Diabetes	No			
Yes		0.095	1.10 (0.86–1.42)	0.459
Smoking history	Never smoking			
Current smoking		−0.251	0.78 (0.55–1.11)	0.164
Ever smoking		−0.250	0.78 (0.52–1.17)	0.230
Drinking history	Never drinking			
Current drinking		0.011	1.01 (0.72–1.42)	0.948
Ever drinking		0.126	1.13 (0.70–1.85)	0.611
History of angina pectoris	No			
Yes		0.102	1.11 (0.59–2.10)	0.754
History of myocardial infarction	No			
Yes		0.634	1.89 (0.34–10.38)	0.466
Total cholesterol TC, mmol/L		−0.019	0.98 (0.89–1.08)	0.700
Triglyceride TG, mmol/L		−0.012	0.99 (0.90–1.08)	0.797
Creatinine, μmol/L		−0.003	1.00 (0.99–1.00)	0.334
BRI		0.039	1.04 (0.93–1.17)	0.509

(1) The adjusted factors are age, sex, BMI, hypertension, diabetes, smoking history, drinking history, angina pectoris and myocardial infarction history, TG, TC and creatinine. (2) The missing values annotated in Table 2 were excluded from the multifactor analysis.

**Table 4:** The association of BRI quartile and cognitive impairment in the multivariate analysis.

Characteristics	Reference	*β*	OR (95%CI)	*P*
Gender	Man			
Woman		0.498	1.65(1.17–2.33)	0.005
Age, years old	<65 years old			
≥65 years old		0.714	2.04(1.68–2.49)	<0.001
BMI grouping	Normal or underweight			
Overweight		−0.128	0.88(0.67–1.16)	0.361
Obesity		−0.435	0.65(0.44–0.95)	0.024
Hypertension	No			
Yes		0.375	1.46(1.14–1.87)	0.003
Diabetes	No			
Yes		0.095	1.10(0.85–1.42)	0.462
Smoking history	Never smoking			
Current smoking		−0.276	0.76(0.53–1.08)	0.127
Ever smoking		−0.263	0.77(0.51–1.16)	0.210
Drinking history	Never drinking			
Current drinking		0.043	1.04(0.74–1.47)	0.802
Ever drinking		0.181	1.20(0.74–1.95)	0.467
History of angina pectoris	No			
Yes		0.068	1.07(0.57–2.03)	0.834
History of myocardial infarction	No			
Yes		0.626	1.87(0.35–10.14)	0.468
Total cholesterol TC, mmol/L		−0.014	0.99(0.90–1.09)	0.781
Triglyceride TG, mmol/L		−0.007	0.99(0.91–1.09)	0.873
Creatinine, μmol/L		−0.003	1.00(0.99–1.00)	0.376
BRI quartile	Q1			
Q2		−0.373	0.69 (0.51–0.94)	0.017
Q3		−0.141	0.87 (0.62–1.23)	0.425
Q4		−0.037	0.96 (0.65–1.44)	0.855

(1) The adjusted factors are age, sex, BMI, hypertension, diabetes, smoking history, drinking history, angina pectoris and myocardial infarction history, TG, TC and creatinine. (2) The missing values annotated in Table 2 were excluded from the multifactor analysis.

#### 3.3.1 Model 1

After adjusting for multiple confounding variables, the analysis revealed that women had a 1.59 times higher risk of cognitive impairment compared to men (OR: 1.59, 95% CI: 1.12–2.25, *p* = 0.009). Participants aged 65 and older were found to have a 2.05 times greater risk of cognitive impairment compared to those under 65 (OR: 2.05, 95% CI: 1.69–2.50, *p* < 0.001). Interestingly, obese individuals had a 36% lower risk of cognitive impairment compared to non-obese participants (OR: 0.64, 95% CI: 0.43–0.94, *p* = 0.022). Additionally, hypertensive participants had a 1.45 times greater risk of cognitive impairment compared to those without hypertension (OR: 1.45, 95% CI: 1.13–1.85, *p* = 0.004). However, BRI as a continuous variable did not show a significant association with cognitive impairment (*p* = 0.509; Table 3).

#### 3.3.2 Model 2

In contrast, when BRI was categorized into quartiles, we found that participants in the second quartile of BRI (3.5897 ≤ BRI < 4.4898) had a 31% lower risk of cognitive impairment compared to those in the first quartile (BRI < 3.5897) (OR: 0.69, 95% CI: 0.51–0.94, *p* = 0.017). This association suggests that there may be a non-linear relationship between BRI and cognitive impairment, with certain BRI ranges potentially offering protective effects (Table 4).

### 3.4 Subgroup analysis of BRI and cognitive impairment

**Figure 2:**
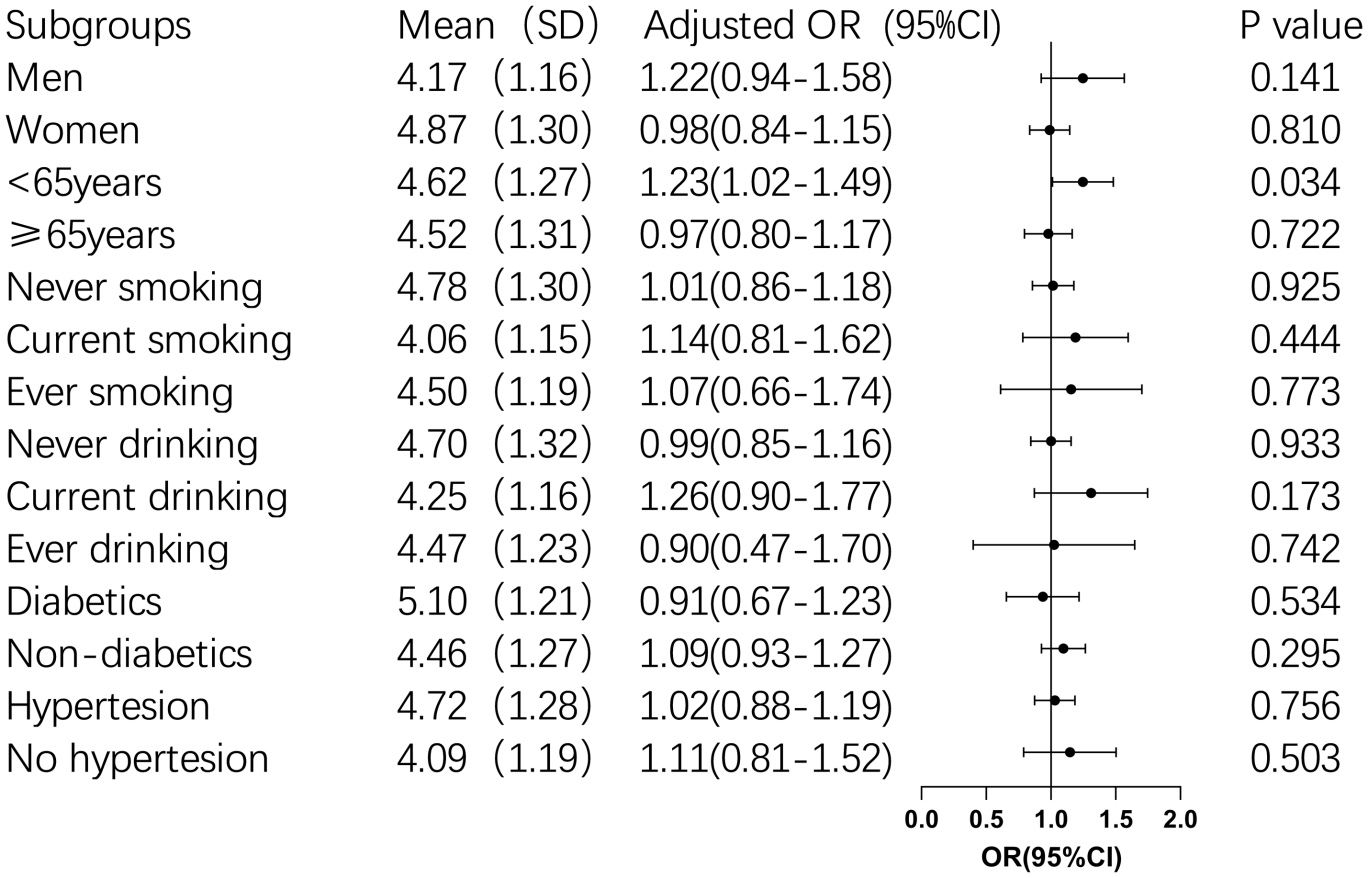
Subgroup Analysis of BRI and Cognitive Impairment.

Subgroup analysis was conducted for BRI continuous variables with insignificant results of multi-factor logistic regression to further explore their correlation. Subgroup analysis revealed that for participants younger than 65 years old, each unit increase in BRI was associated with a 23% higher risk of cognitive impairment (OR: 1.23, 95% CI: 1.02–1.49, *p* = 0.034). Furthermore, BRI was found to interact significantly with age, sex, and hypertension (*p* < 0.05), suggesting these factors jointly influence the risk of cognitive impairment (Table 4 and Figure 2).

### 3.5 Non-linear relationship between BRI and MMSE score

**Figure 3:**
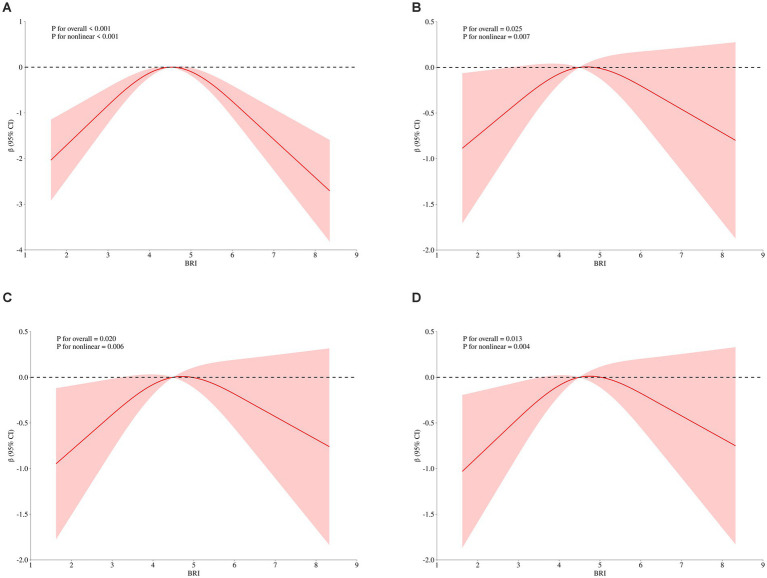
Nonlinear Relationship Between BRI and MMSE Score.

**Figure 4:**
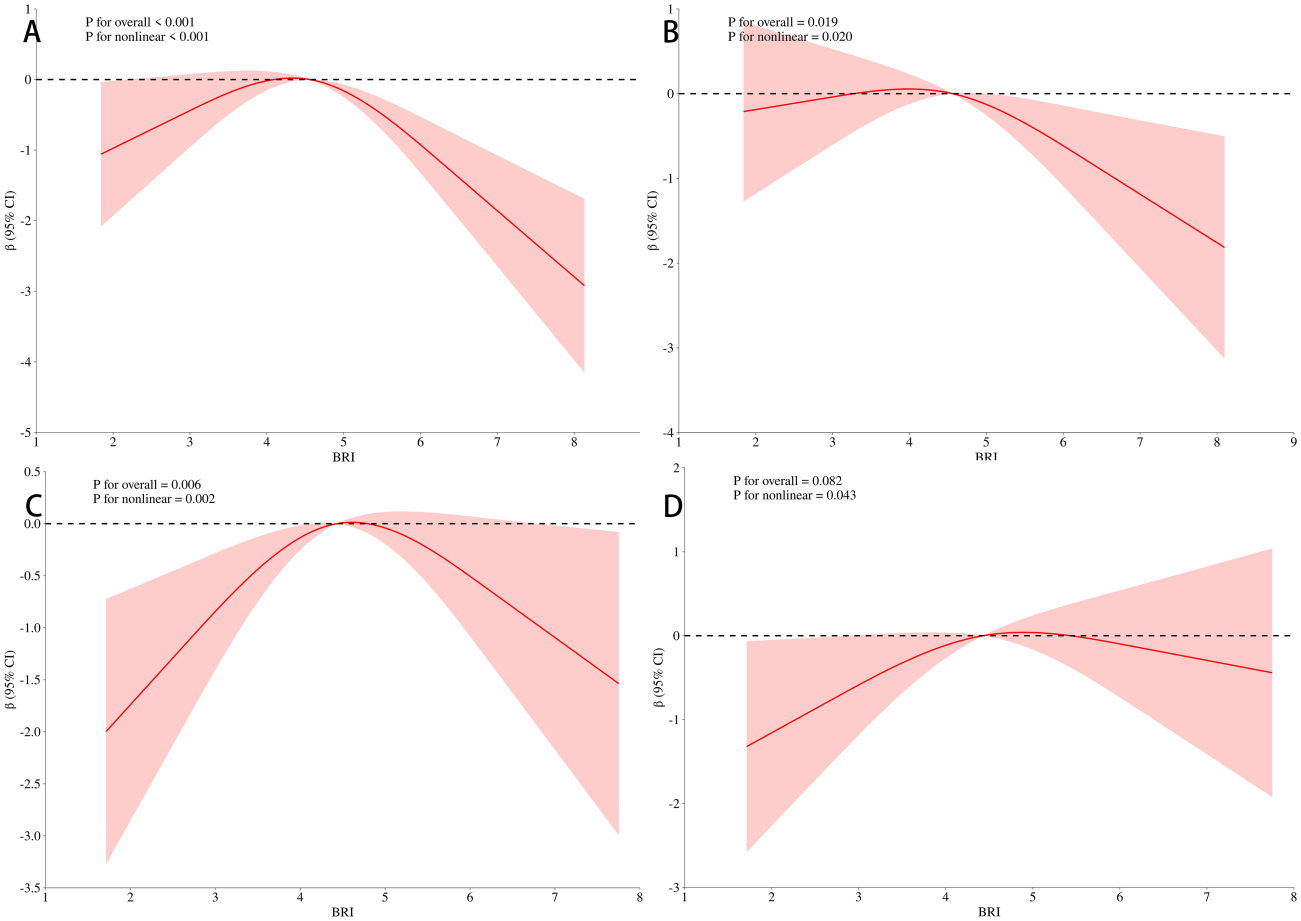
Association of BRI with MMSE scores in the stratified analysis. Panel **(A)** showed a significant inverse U-shaped correlation (*P* < 0.001) after univariate analysis under 65 years old. Panel **(B)** showed that the curve inflection point of multi-factor correction was 4.56 (*P* = 0.019), after adjustment for all the above factors under 65 years old. Panel **(C)** showed that the univariate inverse U-shaped association was significant in people aged 65 years and older (*P* = 0.006). Panel **(D)** showed the significance disappeared after adjusting for multiple factors (*P* = 0.082), the curve inflection point was 4.29 in people aged 65 years and older.

To explore the dose–response relationship between BRI and cognitive function, we analyzed the non-linear relationship between BRI and MMSE score using restricted cubic splines. The analysis revealed an inverse U-shaped relationship between BRI and MMSE score (Figure 3A). After adjusting for gender, age, educational level, and BMI, the negative correlation between BRI and MMSE score increased when BRI exceeded 4.49, while below this threshold, the correlation weakened (*p* = 0.007; Figure 3B). These results remained significant after further adjustments for smoking, drinking, diabetes, hypertension, myocardial infarction, and angina (*p* = 0.006; Figure 3C). Additional adjustments for laboratory indices such as TG and TC showed that the weakest negative correlation with MMSE occurred near a BRI of 4.49 (*p* = 0.004; Figure 3D), confirming the inverted U-shaped relationship. We further stratified by age, and found that BRI and MMSE scores of participants under 65 years old showed a significant inverse U-shaped correlation (*p* < 0.05) after univariate factor (Figure 4A) and adjustment for all the above factors (Figure 4B), and the curve inflection point of multi-factor correction was 4.56. The univariate inverse U-shaped association was significant (*p* = 0.043; Figure 4C) in people aged 65 years and older, and the significance disappeared after adjusting for multiple factors (*p* = 0.082; Figure 4D), and the curve inflection point was 4.29 (Table 5).

**Table 5:** Subgroup analysis of BRI and cognitive impairment.

Subgroups	Adjust OR (95%CI)	*p*-value	*P* for interaction
Gender			<0.001
Man	1.22 (0.94–1.58)	0.141	
Woman	0.98 (0.84–1.15)	0.810	
Age			<0.001
<65 years old	1.23 (1.02–1.49)	0.034	
≥65 years old	0.97 (0.80–1.17)	0.722	
Smoking history			0.122
Never smoking	1.01 (0.86–1.18)	0.925	
Current smoking	1.14 (0.81–1.62)	0.444	
Ever smoking	1.07 (0.66–1.74)	0.773	
Drinking history			0.659
Never drinking	0.99 (0.85–1.16)	0.933	
Current drinking	1.26 (0.90–1.77)	0.173	
Ever drinking	0.90 (0.47–1.70)	0.742	
Diabetes			0.822
Diabetes	0.91 (0.67–1.23)	0.534	
Non-diabetics	1.09 (0.93–1.27)	0.295	
Hypertension			0.024
Hypertension	1.02 (0.88–1.19)	0.756	
Non-hypertension	1.11 (0.81–1.52)	0.503	

The adjusted factors are age, sex, BMI, hypertension, diabetes, smoking history, drinking history, angina pectoris and myocardial infarction history, TG, TC and creatinine. All subgroups are adjusted for all factors except the grouping variables.

## 4 Discussion

The primary objective of this study was to investigate the relationship between BRI and cognitive impairment in a low-income, low-education population. Given the high prevalence of cognitive impairment, we aimed to explore whether BRI could serve as an independent predictor of cognitive decline. Our study is the first to assess the dose–response relationship between BRI, MMSE scores, and cognitive impairment in a rural Chinese population. The key findings reveal that women, individuals aged 65 and above, and those with hypertension are more likely to experience cognitive decline. Notably, obese participants exhibited a lower risk of cognitive impairment compared to those with normal or underweight status. Furthermore, individuals in the second BRI quartile demonstrated a lower risk of cognitive impairment than those in the first quartile. Although no direct correlation between BRI and cognitive impairment was found overall, BRI was shown to interact significantly with age, gender, and hypertension, jointly influencing cognitive outcomes.

The relationship between BRI, a measure of obesity, and cognitive function remains controversial. Obesity has been linked to cognitive impairment throughout adulthood and is known to increase the risk of dementia in later life (Leigh and Morris, 2020). In the brain, obesity may result in various types of damage, including oxidative stress, inflammation, protein aggregation, mitochondrial dysfunction, hormonal imbalances, IR, blood–brain barrier damage, and disruptions in synaptic plasticity and neurogenesis, all of which can contribute to cognitive decline and neuronal death (Neto et al., 2023). Despite this, most cross-sectional studies have not demonstrated a significant relationship between BRI and cognition. The weight-adjusted waist circumference index (WWI) is linearly related to the occurrence of AD and other dementias, but they found no association between BRI and dementia in rural China (Wang et al., 2023). Similarly, a cross-sectional study from Iran indicated that increased BMI, WC, and waist-to-hip ratio were associated with a reduced risk of cognitive decline, yet no significant correlation was observed between BRI and any cognitive test (Ramezani Kashal et al., 2024). Among non-demented multi-ethnic Asians with type 2 diabetes, while univariate analysis found a significant relationship between BRI and cognition, multivariate analysis revealed no such correlation (Moh et al., 2020). In contrast, a study conducted in a Taiwanese population over 60 years old reported a significant association between BRI and reduced MMSE scores, though it did not directly address the relationship between BRI and cognitive impairment (Huang et al., 2022). Zhang et al. (2024) also reported a significant correlation between BRI level and cognitive performance in people over 65 years of age in the United States, with higher BRI significantly associated with lower Digit Symbol Substitution Test (DSST) scores measuring cognition, and a stronger negative association in men. Our findings align with some aspects of previous research, as we did not observe a direct relationship between BRI and cognitive impairment across the entire sample. However, we did find that participants in the second quartile of BRI had a lower risk of cognitive impairment compared to those in the first quartile, though this effect was not observed in the higher quartiles. Further analysis revealed that BRI interacts significantly with age, gender, and hypertension, highlighting its varying influence on cognitive impairment across different subgroups. Specifically, the correlation between BRI and cognitive decline was stronger in women than in men, and more pronounced in participants younger than 65 years old, while the predictive power of BRI diminished in older individuals. Unlike Zhang et al. (2024), we chose MMSE, a more comprehensive scale for assessing cognitive impairment, rather than DSST, but the results may be different due to the influence of participants’ self-reported education level and the differences in age and region of the study population selected by Zhang’s team.

A key difference between our study and previous research (Huang et al., 2022), is the recognition that the relationship between BRI and MMSE scores is not strictly linear. Using a RCS model, we demonstrated that the association between BRI and cognitive decline is more complex, exhibiting an inverted U-shaped correlation. The weakest relationship between BRI and MMSE scores occurred when BRI was around 4.49, suggesting that both low and high BRI values may have differing impacts on cognitive outcomes. Unlike earlier studies, we included a wider age range. At the same time, the age stratification results still showed an inverted U-shaped curve, and it was obvious in people under 65 years old, which allowed us to identify that BRI may serve as a better predictor of cognitive function among younger individuals.

The relationship between cognitive impairment and the traditional obesity measure was well-established. A cross-sectional study found that being overweight was associated with a reduced risk of cognitive impairment among the elderly in China (Hou et al., 2019). Similarly, as BMI increased, the risk of cognitive decline decreased among elderly individuals in Iran (Ramezani Kashal et al., 2024). Most prospective studies support the view that being underweight is linked to cognitive decline (Ren et al., 2021; Zhang et al., 2022; Wu et al., 2021; Dong et al., 2023), whereas being overweight or obese is often considered protective against cognitive impairment (Zhang et al., 2022; Wu et al., 2021; Dong et al., 2023; Liang et al., 2022). A study highlighted the age-related effects of BMI on cognition, noting that before the age of 65, a higher BMI was associated with lower cognitive ability, but after 65, the relationship reversed, with higher BMI correlating with better cognitive outcomes (Crane et al., 2023). In line with these studies, we found that individuals with cognitive impairment had a lower BMI compared to those without cognitive impairment, and obese individuals had a lower risk of cognitive impairment compared to those with normal or underweight status. These variations may be explained by the complex interplay between BMI, age, and gender. The impact of BMI on cognition appears to shift depending on the demographic characteristics of the study population, which may account for the differing conclusions across studies. Our study is the first to report that the association between BRI and cognition is more significant in middle-aged and young adults, while the relationship is weakened in older adults.

In addition to BMI, the risk of cognitive impairment is also influenced by gender, age, and hypertension. Numerous cross-sectional studies have shown that the prevalence of cognitive impairment is significantly higher in women compared to men, particularly among middle-aged and elderly populations (Miyawaki and Liu, 2019; Roh et al., 2021; Han et al., 2022; Wang et al., 2020). Furthermore, a meta-analysis demonstrated that individuals with hypertension have a notably high prevalence of cognitive impairment (Qin et al., 2021). Cross-sectional studies further support the role of hypertension as a significant risk factor for cognitive impairment (Chen et al., 2022), making it a valuable predictor of cognitive decline in rural populations (Bao et al., 2022). Our findings was similar to these previous studies, as we observed that the risk of cognitive impairment increases with age, and women are more likely to experience cognitive impairment compared to men. Additionally, there is a strong correlation between hypertension and cognitive impairment, with hypertensive individuals facing a greater risk of cognitive decline. Notably, our study is the first to identify that BRI interacts with age, gender, and hypertension, jointly influencing the risk of cognitive impairment. This interaction enhances BRI’s utility as an independent predictor of cognitive decline.

This study has several limitations. First, the sample population was drawn exclusively from rural areas of Tianjin, China, which may limit the generalizability of the findings to urban populations or those in other regions. Future studies should include more diverse populations to validate these results. Second, as a cross-sectional study, we were unable to establish a causal relationship between BRI and cognitive impairment. Longitudinal studies would be necessary to explore the temporal relationship and potential causality. Third, cognitive impairment was assessed using the MMSE, which, while widely used, may not capture all aspects of cognitive function. Incorporating more comprehensive cognitive assessments in future studies could provide a more complete evaluation. Fourth, lifestyle factors such as diet, exercise, and medication history were not considered, which may have influenced the relationship between BRI and cognition. Future research should account for these confounding factors to strengthen the reliability of the findings. Finally, education years were self-reported, which may introduce reporting bias. Using objective data from medical or educational records in future studies could reduce this potential bias.

## 5 Conclusion

This study highlights the complex relationship between BRI and cognitive impairment, demonstrating that BRI, when considered alongside factors such as age, gender, and hypertension, can provide valuable insights into cognitive risk. The findings suggest that BRI may serve as an independent predictor of cognitive decline, particularly in younger individuals and women, offering clinicians a potential tool for early identification of those at risk. Patients can slow cognitive decline by early detection of cognitive impairment through easy-to-measure indicators such as BRI and timely intervention. Healthcare providers can incorporate BRI into routine assessments to enhance screening practices, especially in high-risk populations. Early intervention and risk stratification based on BRI may reduce the economic burden associated with long-term care for dementia, thereby improving public health outcomes.

## Data Availability

The raw data supporting the conclusions of this article will be made available by the authors, without undue reservation.
